# Morbid Obesity as a Risk Factor for Hospitalization and Death Due to 2009 Pandemic Influenza A(H1N1) Disease

**DOI:** 10.1371/journal.pone.0009694

**Published:** 2010-03-15

**Authors:** Oliver W. Morgan, Anna Bramley, Ashley Fowlkes, David S. Freedman, Thomas H. Taylor, Paul Gargiullo, Brook Belay, Seema Jain, Chad Cox, Laurie Kamimoto, Anthony Fiore, Lyn Finelli, Sonja J. Olsen, Alicia M. Fry

**Affiliations:** 1 Division of Emerging Infections and Surveillance Services, Centers for Disease Control and Prevention, Atlanta, Georgia, United States of America; 2 Influenza Division, Centers for Disease Control and Prevention, Atlanta, Georgia, United States of America; 3 Division of Nutrition and Physical Activity and Obesity, Centers for Disease Control and Prevention, Atlanta, Georgia, United States of America; 4 Division of Bacterial Diseases, Centers for Disease Control and Prevention, Atlanta, Georgia, United States of America; Institute of Preventive Medicine, Denmark

## Abstract

**Background:**

Severe illness due to 2009 pandemic A(H1N1) infection has been reported among persons who are obese or morbidly obese. We assessed whether obesity is a risk factor for hospitalization and death due to 2009 pandemic influenza A(H1N1), independent of chronic medical conditions considered by the Advisory Committee on Immunization Practices (ACIP) to increase the risk of influenza-related complications.

**Methodology/Principal Findings:**

We used a case-cohort design to compare cases of hospitalizations and deaths from 2009 pandemic A(H1N1) influenza occurring between April–July, 2009, with a cohort of the U.S. population estimated from the 2003–2006 National Health and Nutrition Examination Survey (NHANES); pregnant women and children <2 years old were excluded. For hospitalizations, we defined categories of relative weight by body mass index (BMI, kg/m^2^); for deaths, obesity or morbid obesity was recorded on medical charts, and death certificates. Odds ratio (OR) of being in each BMI category was determined; normal weight was the reference category. Overall, 361 hospitalizations and 233 deaths included information to determine BMI category and presence of ACIP-recognized medical conditions. Among ≥20 year olds, hospitalization was associated with being morbidly obese (BMI≥40) for individuals with ACIP-recognized chronic conditions (OR = 4.9, 95% CI 2.4–9.9) and without ACIP-recognized chronic conditions (OR = 4.7, 95%CI 1.3–17.2). Among 2–19 year olds, hospitalization was associated with being underweight (BMI≤5^th^ percentile) among those with (OR = 12.5, 95%CI 3.4–45.5) and without (OR = 5.5, 95%CI 1.3–22.5) ACIP-recognized chronic conditions. Death was not associated with BMI category among individuals 2–19 years old. Among individuals aged ≥20 years without ACIP-recognized chronic medical conditions death was associated with obesity (OR = 3.1, 95%CI: 1.5–6.6) and morbid obesity (OR = 7.6, 95%CI 2.1–27.9).

**Conclusions/Significance:**

Our findings support observations that morbid obesity may be associated with hospitalization and possibly death due to 2009 pandemic H1N1 infection. These complications could be prevented by early antiviral therapy and vaccination.

## Introduction

Since April 2009, the 2009 pandemic influenza A(H1N1) virus, a novel influenza virus with a combination of gene segments not previously reported in swine or human influenza viruses, has spread worldwide and caused epidemics of disease. [1], [2] Early reports suggest that persons with chronic medical conditions previously recognized as increasing the risk for complications due to seasonal influenza also have a higher risk of complications due to 2009 pandemic influenza A(H1N1) infection. [3], [4] Consequently, the Advisory Committee on Immunization Practices (ACIP) included individuals with these recognized chronic medical conditions in their priority groups for receipt of the new 2009 pandemic influenza A(H1N1) vaccine. [5]

Preliminary data from the United States and abroad suggest that obesity (body mass index, BMI≥30 kg/m^2^) and morbid obesity (BMI≥40 kg/m^2^) is disproportionately represented among hospitalizations, intensive care admissions, and deaths from 2009 pandemic influenza A(H1N1). [6], [7], [8], [9] We explored the hypothesis that BMI is a risk factor for hospitalization and death due to pandemic influenza A(H1N1), independent of ACIP-recognized chronic medical conditions.

## Methods

We used three datasets: patients hospitalized with 2009 pandemic influenza A(H1N1), deaths from 2009 pandemic influenza A(H1N1), and the National Health and Nutrition Examination Survey (NHANES), a representative survey of the general U.S. population. For each dataset we defined individuals according to BMI category (see below), presence of ACIP-recognized chronic medical conditions (defined for this analysis as cardiovascular disease, pulmonary disease, liver disease, cancer, and diabetes) and age group. We did not include neurologic, neuromuscular, hematological, non-diabetes metabolic disorders or immunosuppression as ACIP-recognized chronic medical conditions in this analysis due to lack of this information from NHANES. We used two age groups, 2–19 years and ≥20 years: dividing the ≥20 year old age group into additional age categories resulted in unreliable variance estimates when we stratified by BMI category and presence of ACIP-recognized chronic medical conditions due to zero observations in primary sampling units of the NHANES survey.

For hospitalized patients and NHANES participants we used BMI [height (m)/weight (kg)^2^] as an index of relative weight. For individuals aged 2–19 years, we calculated age- and sex-specific BMI z-scores and used CDC 2000 growth charts to define BMI category, where underweight was <5^th^ percentile, normal weight 5–84^th^ percentile, overweight 85–94^th^ percentile, and obese was ≥95^th^ percentile. [10], [11], [12] For individuals ≥20 years we defined underweight as BMI<18.5, normal weight as 18.5–24.9, overweight as 25.0–29.9, obese as 30.0–39.9, and morbidly obese ≥40.0. [13], [14] For deaths, no data were available on measured height and weight, so we used mention of obesity or morbid obesity within the medical condition section of the reporting form. For all three datasets we excluded individuals <2 years old and pregnant women because obesity definitions are not applicable to them.

We included information on 516 (10%) of 5009 patients hospitalized with 2009 pandemic influenza A(H1N1) in the United States between May 1–July 12, 2009. These patients were a convenience sample of all hospitalizations reported to CDC from 24 states as part of prospective surveillance project, as described previously, [15] for which state health departments abstracted information from medical charts using a standard case form. We were provided with additional reports of hospitalizations from New York City, Wisconsin, and Texas, that were not included in the surveillance project, but had been collected by health departments using the same or similar abstraction forms during the same time period. If height and weight were not reported in data collected for the prospective study, we attempted to contact patients for this information. For a minority of patients for whom we had weight but not height data, we estimated their obesity category by assuming that their height was not less than the 5^th^ percentile or greater than the 95^th^ percentile of the population (see Supplement S1). We included information on deaths reported to CDC from April 27 to July 23, 2009. State health departments collected information from medical charts, medical examiner reports, and death certificates, using a standard data collection form. All hospitalizations and deaths included in our analyses were confirmed to have pandemic H1N1 infection by real-time reverse transcription polymerase chain reaction (rRT-PCR). [16] We estimated the proportion of the U.S. population in different BMI categories from NHANES 2003–4 and 2005–6. NHANES methodology has been described previously. [17]

For each dataset, we calculated the proportion of individuals in each BMI category by age group (2–19 years and ≥20 years). We stratified our analysis by presence or absence of an ACIP-recognized chronic medical condition, which is likely to confound the relationship between BMI category and hospitalization or death due to 2009 pandemic A(H1N1) influenza. We calculated the ratio of odds (odds ratio, OR) of being in each BMI category, where the normal weight category was the reference category, for hospitalizations and deaths (cases) compared to the U.S. population (cohort). Because we compared cases to the cohort of the U.S. population selected before the time period during which cases could occur (i.e., a case-cohort design), the OR estimates the risk ratio of hospitalization or death by BMI category. [18] To estimate 95% confidence intervals (95%CI) and p-values of the odds ratios we used the Taylor series linearization method to combine the variance estimate of the log-odds of exposure among cases (hospitalizations or deaths) with a variance of the log-odds of exposure among the population as estimated using NHANES. [19], [20] For hospitalized patients, we conducted a sensitivity analysis to consider how robust our analysis was with regard to adjustment of age among ≥20 year olds, by creating a logistic regression model for each BMI category with Normal weight as the reference category, where hospitalization was the dependent variable and age group (20.0–35.4, 35.5–45.9, 46.0–54.9, ≥55.0 years) was the independent variable (see Supplement S1). We conducted a further sensitivity analysis of our treatment of missing BMI data (see Supplement S1). We also repeated the analysis for hospitalized patients after changing the comparison group to a cohort of the U.S. population that had been admitted to hospital for any reason in a 12 month period, as estimated by NHANES. The rationale for this additional analysis was to assess whether any observed association between BMI category and hospitalization was due to more frequent hospitalization among obese and morbidly obese individuals. We used SAS 9.2 for all analyses.

The investigation of 2009 pandemic influenza A(H1N1) infections was deemed public health surveillance, not human subjects research and therefore did not require Institutional Review Board review.

## Results

Between April and July 2009, we identified 565 hospitalized patients with confirmed 2009 pandemic H1N1 infection; we excluded 51 patients who were pregnant and 77 who were <2 years old. Of the remaining 437 patients, 283 (65%) had both measured height and weight which we used to calculate BMI. In addition, we estimated BMI category for 78 (19%) patients who had data on measured weight but not measured height; 52 (27%, n = 52/191) were patients aged 2–19 years, of which 19% were estimated to be underweight and 23% were estimated to be obese; 26 (11%, n = 26/246) were patients ≥20 years, of which 42% were estimated to be obese or morbidly obese. Our final hospitalized cohort included 361 patients assigned to a BMI category.

There were 304 deaths among persons with 2009 pandemic influenza A(H1N1) reported to CDC; 16 pregnant women and eight children <2 years old were excluded. Of the remaining 280 deaths, 10 (4%) were missing data about chronic medical conditions and 37 (13%) about obesity, leaving 233 deaths in our analysis: 31 were 2–19 years old and 202 were ≥20 years old.

In the U.S. population aged 2–19 years, the prevalence of ACIP-recognized chronic medical conditions increased with increasing BMI category: 16.4% were obese and 20.7% had ACIP-recognized chronic medical conditions (Table 1). In comparison, among hospitalized patients 2–19 years old, 25.5% were obese, of whom 53.7% had ACIP-recognized chronic medical conditions. Among deaths in 2–19 year olds, 9.7% were obese, of whom 33.3% had ACIP-recognized chronic medical conditions. In the US population ≥20 years old, 28.0% were obese and 5.4% were morbidly obese; the prevalence of ACIP-recognized chronic medical conditions increased only slightly with increasing BMI category: 40.9% of obese and 51.2% of morbidly obese persons had ACIP-recognized chronic medical conditions. Among hospitalized patients with 2009 pandemic H1N1 infection ≥20 years old, 32.5% were obese and 67.7% of these obese patients had ACIP-recognized chronic medical conditions, while 22.5% were morbidly obese, of whom 77.8% had ACIP-recognized chronic medical conditions. Among deaths in ≥20 year olds, 38.1% were obese and 12.4% were morbidly obese; ACIP-recognized chronic medical conditions were reported for 63.6% of deaths among obese and 56.0% of morbidly obese individuals.

**Table 1:** Number and percent of persons with ACIP-recognized chronic underlying medical conditions, by category of body mass index (BMI).

BMI Category	NHANES 2003–2006 (thousands)*				Hospitalized Patients				Deaths with pandemic H1N1†			
	Individuals	(%)	Chronic Medical Condition**	(%)‡	Individuals	(%)	Chronic Medical Condition**	(%)‡	Individuals	(%)	Chronic Medical Condition**	(%)‡
**2–19 Years Old**												
Underweight	2,359	(3.3)	214	(9.1)	25	(15.5)	14	(56.0)	-	-	-	-
Normal weight	46,187	(64.8)	6,667	(14.4)	72	(44.7)	35	(48.6)	28	(90.3)	8	(28.6)
Overweight	11,022	(15.5)	1,972	(17.9)	23	(14.3)	15	(65.2)	-	-	-	-
Obese	11,660	(16.4)	2,419	(20.7)	41	(25.5)	22	(53.7)	3	(9.7)	1	(33.3)
Total	71,228	(100)			161	(100)			31	(100)	0	
**≥20 Years Old**												
Underweight	3,501	(1.8)	1,248	(35.7)	5	(2.5)	3	(60.0)	-	-	-	-
Normal weight	61,798	(31.4)	18,318	(29.6)	42	(21.0)	24	(57.1)	100	(49.5)	75	(75.0)
Overweight	65,728	(33.4)	21,316	(32.4)	43	(21.5)	28	(65.1)	-	-	-	-
Obese	55,022	(28.0)	22,527	(40.9)	65	(32.5)	44	(67.7)	77	(38.1)	49	(63.6)
Morbidly Obese	10,635	(5.4)	5,450	(51.2)	45	(22.5)	35	(77.8)	25	(12.4)	14	(56.0)
Total	196,684	(100)			200	(100)			202	(100)		

Note: Pregnant women and children <2 not included.

BMI (kg/m^2^) categories for 2–19 year olds based on age- and sex-specific CDC 2000 growth charts: underweight<5^th^ percentile, normal weight 5–84^th^ percentile, overweight 59–94^th^ percentile, obese ≥95^th^ percentile.

BMI (kg/m^2^) categories for ≥20 year olds: underweight<18.5, normal weight 18.5–24.9, overweight 25.0–29.9, obese 30.0–39.9, morbidly obese ≥40.

* National Health and Nutrition Examination Survey (NHANES) 2003–2006.

** For this analysis ACIP-recognized chronic medical conditions include cardiovascular disease, pulmonary disease, liver condition, cancer, and diabetes.

† Underweight, Normal, and overweight categories not separately defined for deaths.

‡ Row percent.

Among patients 2–19 years, there was no statistically significant association between being overweight or obese and hospitalization among those who did or did not have ACIP-recognized chronic medical conditions (Table 2). However, being underweight was associated with hospitalization among patients 2–19 years old with and without ACIP-recognized chronic medical conditions (OR = 12.5, 95%CI 3.4 to 45.5, p<0.001; OR = 5.5, 95%CI 1.3 to 22.5, p<0.001, respectively) (Table 2). There was no association between BMI category and death among persons 2–19 years old (Table 2).

**Table 2:** Odds Ratio of BMI category for patients hospitalized or deaths with 2009 pandemic influenza A(H1N1) compared to the U.S. population, by category of body mass index (BMI) and presence of ACIP-recognized chronic medical conditions*.

BMI Category	Hospitalized Patients with 2009 Influenza A(H1N1)						Deaths with 2009 Influenza A(H1N1) †					
	ACIP-recognized Chronic medical conditions*			No ACIP-recognized Chronic Medical Conditions			ACIP-recognized Chronic medical conditions*			No ACIP-recognized Chronic Medical Conditions		
	OR	95%CI	p-value	OR	95%CI	p-value	OR	95%CI	p-value	OR	95%CI	p-value
**2–19 Years Old**												
Underweight	12.5	3.4 to 45.5	<0.001	5.5	1.3 to 22.5	<0.001						
Normal weight	Ref	-		Ref	-		Ref	-	-	Ref	-	-
Overweight	1.4	0.5 to 4.5	0.52	0.9	0.2 to 5.8	1.0						
Obese	1.7	0.7 to 4.22	0.23	2.2	0.4 to 13.4	0.4	0.5	<0.01 to 235	0.81	0.5	<0.01 to 68	0.81
**≥20 Years Old**												
Underweight	1.8	0.05 to 68	0.74	2.1	0.0 to 219	0.7						
Normal weight	Ref	-		Ref	-		Ref	-	-	Ref	-	-
Overweight	1.0	0.5 to 2.1	0.99	0.8	0.3 to 2.3	0.7						
Obese	1.5	0.8 to 2.8	0.22	1.6	0.7 to 3.7	0.3	1.2	0.7 to 2.1	0.57	3.1	1.5 to 6.6	<0.001
Morbidly Obese	4.9	2.4 to 9.9	<0.001	4.7	1.3 to 17.2	<0.001	1.4	0.3 to 6.0	0.65	7.6	2.1 to 27.9	<0.001

Note: OR, Odds Ratio, CI, confidence interval.

BMI (kg/m^2^) categories for 2–19 year olds based on age- and sex-specific CDC 2000 growth charts: underweight<5^th^ percentile, normal weight 5–84^th^ percentile, overweight 59–94^th^ percentile, obese ≥95^th^ percentile.

BMI (kg/m^2^) categories for ≥20 year olds: underweight<18.5, normal weight 18.5–24.9, overweight 25.0–29.9, obese 30.0–39.9, morbidly obese ≥40.

* For this analysis ACIP-recognized chronic medical conditions include cardiovascular disease, pulmonary disease, liver condition, cancer, and diabetes.

† Underweight, Normal, and overweight categories not separately defined for deaths.

Among hospitalized patients aged ≥20 years with and without ACIP-recognized chronic medical conditions, morbid obesity was the only BMI category statistically associated with hospitalization (OR = 4.9, 95%CI 2.4 to 9.9, p<0.001; OR = 4.7, 95%CI 1.3 to 17.2, p<0.001, respectively). These associations remained after adjustment for age (Supplement S1 Table S3), when we excluded patients with measured weight but no measured height (Supplement S1 Table S1) and when we assumed that all persons with missing height and weight were in the normal weight category (Supplement S1 Table S2). However, the association between morbid obesity and hospitalization among individuals who did not have an ACIP-recognized chronic medical condition was not statistically significant in the final sensitivity analysis (Supplement S1 Table S2).

BMI category was not associated with death among adults who had an ACIP-recognized chronic medical condition. However, death was associated with obesity and morbid obesity among adults who did not have an ACIP-recognized chronic medical condition (OR = 3.1, 95%CI 1.5 to 6.6, p<0.001; OR = 7.6, 95%CI 2.1 to 27.9, p<0.001, respectively) (Table 2).

When we repeated our analysis using the subgroup of the U.S. population that had been hospitalized in the previous year as the comparison group, morbid obesity remained associated with hospitalization among ≥20 year olds with ACIP-recognized chronic medical condition (OR = 4.1, 95%CI 2.0 to 8.7, p<0.001), but not significantly so for patients with no ACIP-recognized chronic medical condition (OR = 4.2, 95%CI 1.0 to 18.0, p = 0.1) (Table 3).

**Table 3:** Odds Ratio of BMI category for patients hospitalized with 2009 pandemic influenza A(H1N1) compared to the U.S. NHANES 2003–2006 population hospitalized for any reason over a 12-month period, by category of body mass index (BMI) and presence of ACIP-recognized chronic medical conditions.

BMI Category	ACIP-recognized Chronic medical conditions*					No ACIP-recognized Chronic medical conditions				
	NHANES 2003–2006 Hospitalized (thousands)*	Patients	OR	95%CI	p-value	NHANES 2003–2006 Hospitalized (thousands)*	Patients	OR	95%CI	p-value
**2–19 Years Old**										
Underweight	†	14				149	11	4.6	0.9 to 24.1	0.1
Normal weight	958	35	Ref	-		2,299	37	Ref	-	
Overweight	268	15	1.5	0.4 to 5.7	0.5	491	8	1.0	0.2 to 6.6	1.0
Obese	225	22	2.7	0.9 to 7.8	0.1	489	19	2.4	0.8 to 7.6	0.1
**≥20 Years Old**										
Underweight	342	3	2.1	0.1 to 84.9	0.7	117	2	5.1	0.0 to 592	0.5
Normal weight	5,866	24	Ref	-		5,374	18	Ref	-	
Overweight	7,180	28	1.0	0.4 to 2.1	0.9	4,709	15	1.0	0.3 to 2.7	0.9
Obese	8,960	44	1.2	0.6 to 2.3	0.6	5,189	21	1.2	0.5 to 3.0	0.7
Morbidly Obese	2,075	35	4.1	2.0 to 8.7	<0.001	708	10	4.2	1.0 to 18.0	0.1

Note: OR, Odds Ratio, CI, confidence interval.

BMI (kg/m^2^) categories for 2–19 year olds based on age- and sex-specific CDC 2000 growth charts: underweight<5^th^ percentile, normal weight 5–84^th^ percentile, overweight 59–94^th^ percentile, obese ≥95^th^ percentile.

BMI (kg/m^2^) categories for ≥20 year olds: underweight<18.5, normal weight 18.5–24.9, overweight 25.0–29.9, obese 30.0–39.9, morbidly obese ≥40.

* For this analysis ACIP-recognized chronic medical conditions include cardiovascular disease, pulmonary disease, liver condition, cancer, and diabetes.

** National Health and Nutrition Examination Survey (NHANES) 2003–2006, respondents who report having been admitted to hospital for any reason in the 12 months prior to interview.

† There were no observations in this strata from the NHANES survey.

## Discussion

Our analysis of early data from the 2009 pandemic in the United States suggests that morbid obesity, but not obesity, was statistically associated with hospitalization from 2009 pandemic H1N1 influenza among adults both who do and do not have chronic medical conditions. These findings are consistent with reports from surveillance data of a high prevalence of morbid obesity among patients hospitalized with 2009 pandemic H1N1 influenza. [6], [7], [8], [9]. Among 2–19 year olds, hospitalization was associated with being underweight, irrespective of presence of ACIP-recognized chronic medical conditions evaluated in this analysis. Additionally, we found that deaths among ≥20 year olds without ACIP-recognized chronic medical conditions were more likely to be obese or morbidly obese individuals.

Previous studies have demonstrated that obesity was associated with an increased number of all cause hospitalization in adults, [21] although there is no information about the role of obesity in severe disease due to seasonal influenza. In our study, the elevated risk for hospitalization due to 2009 pandemic H1N1 infection persisted even when we restricted our analysis to the U.S. population that reported at least one hospitalization in the previous year, suggesting that the observed associations were not because morbidly obese persons routinely experience higher hospitalization rates. Several studies indicate that morbid obesity may plausibly be an independent risk factor for complications due to 2009 pandemic H1N1 disease: morbid obesity is associated with complications in the intensive care unit, including prolonged stay, prolonged ventilation, and death [22], [23] and women with BMI≥30 have a higher likelihood of developing community acquired pneumonia. [24] The risk for hospitalization among persons ≥20 years of age with morbid obesity was similar regardless of the presence of ACIP-recognized chronic medical conditions, suggesting that the effect of morbid obesity is independent of the presence of ACIP-recognized chronic medical conditions.

We expected to see similar associations between deaths and BMI categories that we saw for hospitalizations. That we did not see the same association, especially for obesity and for persons with ACIP-recognized risk factors, may reflect (1) misclassification of BMI categories among deaths which did not have measured BMI or (2) incomplete reporting of ACIP-recognized chronic medical conditions. Due to the possible misclassification of both BMI and underlying conditions among fatalities these results should be interpreted with caution. Additional studies are necessary to better define the risk of BMI category and underlying conditions, and death due to 2009 pandemic H1N1 infection.

Children aged 2–19 years who were obese had an elevated, but not statistically significant, risk of hospitalization due to 2009 pandemic H1N1 infection and no increased risk of death. However, the number of pediatric deaths and hospitalizations were small and confidence intervals were wide. In addition, children aged 2–19 years who were underweight had an elevated significant risk of hospitalization due to 2009 pandemic H1N1 infection. Among the hospitalized children in the underweight BMI category, 20% (n = 3/15) had neuromuscular/neurodevelopment disorders and 13% (n = 2/15) had immunosuppressive conditions. For this analysis, none of these conditions were included as an ACIP-recognized risk group due to lack of similar data from NHANES. Therefore, inclusion of these children in our analysis may in part be responsible for the observation of an increased risk of hospitalization among underweight children. The collection of additional data over time may give further insight into the risk of BMI category and hospitalization or death due to 2009 pandemic H1N1 infection among children.

This analysis of data collected during the first wave of the 2009 pandemic H1N1 epidemic in the United States was exploratory and findings should be considered preliminary until results from larger and more comprehensive studies are available. There are a number of potential limitations of our analysis. Even though our data about hospitalized patients and deaths were convenience samples, they were collected before the first reports were published suggesting that obesity may be associated with severe pandemic H1N1 disease. [4] We used data from NHANES from 2003–2006 to estimate obesity in the U.S. population, which has remained constant over the last few years and so is likely to reflect the prevalence of obesity in 2009 [25]. Moreover, the high response rates for NHANES (the proportion of sampled individuals who were interviewed and had a physical examination was 76% in 2003–4 and 77% in 2005–6) indicate that obesity-dependent selection bias is unlikely. An advantage of NHANES is that the height and weight of respondents is measured. Medical conditions were self-reported and could have been under- or over-reported. It is hard to determine the extent to which this could have introduced systematic bias. Moreover, NHANES does not collect information about immunosuppression and neurologic/neuromuscular and hematological conditions. [5] To reduce the possibility of bias in our comparison, we did not include these conditions in our definition of ACIP-recognized chronic medical conditions for hospitalized patients or deaths. This is likely to have resulted in an underestimation of the prevalence of chronic medical conditions in all of the data sources included in our analysis. While height and weight were incompletely recorded for hospitalized patients, our results were robust to various assumptions about these missing data and we conclude that the observed associations with morbid obesity were unlikely to be due to missing data bias. However, we had no height or weight data for deaths, and our results of the association between BMI category and death should be interpreted with caution. Finally, some of the observed effect may be due to residual confounding by race/ethnicity, which we could not adjust for due to incomplete data for both hospitalizations and deaths. The impact of this potential bias cannot be determined from our data. Differences in obesity prevalence have been observed only between non-Hispanic black and Mexican-American women but not between other racial/ethnic groups or among men. [26]

Our findings suggest that in the United States persons who are morbidly obese, even if they do not have chronic medical conditions recognized by ACIP as increasing the risk of influenza-related complications, may be at increased risk for hospitalization and potentially also for death due to 2009 pandemic H1N1 infection. However, additional studies with a larger sample of patients and appropriate comparison groups are needed to confirm these findings. We found that at least half of Americans who are morbidly obese reported other ACIP-recognized medical conditions and it is possible that some persons who are morbidly obese also have unrecognized chronic medical conditions. Clinicians should evaluate morbidly obese patients with possible influenza illness for the presence of chronic medical conditions and if these conditions are present, or if persons with morbid obesity show signs of lower respiratory tract infection or other signs of severe illness, these patients should receive empiric antiviral treatment as early as possible. [27] In addition, as vaccine becomes available these persons will likely benefit from vaccination with the 2009 H1N1 vaccine.

## Supporting Information

Supplement S1 Morbid Obesity as a Risk Factor for Hospitalization and Death due to 2009 Pandemic Influenza A(H1N1) Disease.
(0.11 MB DOC)
