# Supplementary material for: Morbid Obesity as a Risk Factor for Hospitalization and Death Due to 2009 Pandemic Influenza A(H1N1) Disease
Source: PLoS One. 2010 Mar 15;5(3):e9694. doi: 10.1371/journal.pone.0009694 (PMC2837749; doi:10.1371/journal.pone.0009694)
Supplement: Supplement S1 — Morbid Obesity as a Risk Factor for Hospitalization and Death due to 2009 Pandemic Influenza A(H1N1) Disease. (0.11 MB DOC) [file pone.0009694.s001.doc]

**Supplement S1**

Morbid Obesity as a Risk Factor for Hospitalization and Death due to 2009 Pandemic Influenza A(H1N1) Disease

**Estimating BMI category**

There were 78 hospitalized patients (52 were 2-19 years old and 26 were ≥20 years old) for whom we had measured weight but no measured height. We therefore estimated which BMI category they were most likely to belong by using a procedure that favored allocation to the normal weight (BMI 18.5 to 24.9) category. We used this conservative approach so that our effect estimates (odds ratios) would not be biased away from 1.0 when using the normal-weight category as the referent group.

Patients ≥ 20 years old

- For every individual we calculated an estimated height as height=√(weight/BMI) based on the BMI cut-points between categories: 18.5, 25, 30, and 40. For a given weight, the estimated height would decrease as the BMI cut-point increases. For example, a weight of 79 kg would correspond to a height of 2.07 m if the BMI were 18.5 kg/m^2^ [18.5=79/2.07^2^], but would correspond to a height of 1.78 m if the BMI were 25 kg/m^2^.
- At each cut-point we used the sex-specific height distribution of the U.S. population (derived from NHANES) to calculate the probability that an adult in the U.S. was likely to be taller than the estimated height of the hospitalized subject.
- We considered subjects as underweight if, at the normal/overweight (BMI=25) cut point, the subject’s estimated height was below the 5^th^ percentile of the U.S. population; none of the adults were underweight.
- For the normal/overweight (BMI=25), overweight/obese (BMI=30) or obese/morbidly (BMI=40) cut points, we selected the cut point at which the height of the individual was below the sex-specific 95th percentile of height in the United States. For example, a male subject with a weight of 254.5 kg had an estimated height of 2.15 m (at BMI=25), 1.96 m (BMI=30) and 1.70 m (BMI=40). Only this last height (1.7 m) was below the 95th percentile of the U.S. population and we therefore classified him as obese. Only 2 subjects (both women, with weights of 123 and 133 kg) were classified as morbidly obese

Patients 2-19 years old

- For every individual we calculated an estimated height as height=√ (weight/BMI), where BMI was age- and sex-specific as defined by the CDC 2000 growth charts.
- We set the BMI cut-points between categories as: BMI=5^th^ percentile for underweight/normal, BMI=85^th^ percentile for normal/overweight, and BMI=95^th^ percentile for overweight/obese.
- We selected the lowest BMI cut point where the estimated height fell within the 5^th^ and 95^th^ percentiles of the age- and sex-specific height distribution from the 2000 CDC growth charts.

**Sensitivity analysis**

We re-ran our analysis of hospitalized patients excluding the 78 patients for whom we estimated BMI category (Table S1). The odds ratios (OR) for being underweight among hospitalized patients 2-19 years old, with and without ACIP-recognized chronic medical conditions, were slightly attenuated but remained statistically significant. The ORs for being morbidly obese among hospitalized patients ≥20 years old, with and without ACIP-recognized chronic medical conditions, increased slightly and remained statistically significant.

We then repeated our analysis including the 78 patients with estimated weight categories, but this time assuming that patients with missing height and weight information were normal weight (Table S2). This assumption reflects a scenario whereby the missing data caused the greatest possible bias of the observed odds ratios away from the null. For patients 2-19 years old with ACIP-recognized chronic medical conditions, the elevated odds ratio for being underweight was attenuated slightly and remained statistically significant. For patients 2-19 years old with no ACIP-recognized chronic medical conditions, the odds ratio for being underweight was also attenuated slightly, but was no longer statistically significant. Similarly, for patients ≥20 years old with ACIP-recognized chronic medical conditions, the elevated odds ratio for being morbidly obese was attenuated but still statistically significant whereas patients ≥20 years old with no ACIP-recognized chronic medical conditions, the odds ratio for being morbidly obese was only slightly elevated and no longer statistically significant.

**Table S1. Odds Ratio for patients hospitalized with 2009 pandemic influenza A(H1N1), where only those with measured height and weight data were included, compared to the U.S. population, by weight category and presence of ACIP-recognized chronic medical conditions***

|  | **ACIP-recognized Chronic medical conditions*** | | | | |  | **No ACIP-recognized Chronic medical conditions** | | | | |
| --- | --- | --- | --- | --- | --- | --- | --- | --- | --- | --- | --- |
|  | US Population (thousands) | Patients** | OR | 95%CI | p-value |  | US Population (thousands) | Patients** | OR | 95%CI | p-value |
|  |  |  |  |  |  |  |  |  |  |  |  |
| **2-19 Years Old** |  |  |  |  |  |  |  |  |  |  |  |
| Underweight | 214 | 7 | 9.9 | 1.6 to 61.4 | <0.001 |  | 2,146 | 8 | 5.5 | 1.0 to 28.6 | <0.001 |
| Normal weight | 6,667 | 22 | Ref | - |  |  | 39,520 | 27 | Ref | - |  |
| Overweight | 1,972 | 13 | 2.0 | 0.6 to 6.3 | 0.2 |  | 9,050 | 3 | 0.5 | <0.01 to 21.1 | 0.7 |
| Obese | 2,419 | 15 | 1.9 | 0.1 to 25.3 | 0.6 |  | 9,241 | 14 | 2.2 | 0.7 to 6.8 | 0.2 |
|  |  |  |  |  |  |  |  |  |  |  |  |
| **≥20 Years Old** |  |  |  |  |  |  |  |  |  |  |  |
| Underweight | 1,248 | 3 | 2.6 | 0.1 to 62.5 | 0.6 |  | 2,253 | 1 | 1.6 | 0.0 to 2529.1 | 0.9 |
| Normal weight | 18,318 | 17 | Ref | - |  |  | 43,480 | 12 | Ref | - |  |
| Overweight | 21,316 | 24 | 1.2 | 0.6 to 3.0 | 0.4 |  | 44,412 | 15 | 1.2 | 0.4 to 3.4 | 0.7 |
| Obese | 22,527 | 41 | 2.0 | 1.0 to 3.9 | 0.1 |  | 32,495 | 17 | 1.9 | 0.7 to 5.0 | 0.2 |
| Morbidly Obese | 5,450 | 35 | 6.9 | 3.3 to 14.4 | <0.001 |  | 5,185 | 9 | 6.3 | 1.7 to 23.8 | <0.001 |

Note: OR, Odds Ratio, CI, confidence interval

BMI (kg/m^2^) categories for 2-19 year olds based on age- and sex-specific CDC 2000 growth charts: underweight<5^th^ percentile, normal weight 5-84^th^ percentile, overweight 59-94^th^ percentile, obese ≥95^th^ percentile.

BMI (kg/m^2^) categories for ≥20 year olds: underweight<18.5, normal weight 18.5-24.9, overweight 25.0-29.9, obese 30.0-39.9, morbidly obese ≥40.

* For this analysis ACIP-recognized chronic medical conditions include cardiovascular disease, pulmonary disease, liver condition, cancer, and diabetes

** Patients only included if they had measured height and weight data.

**Table S2. Odds Ratio for patients hospitalized with 2009 pandemic influenza A(H1N1), where those with no measured height and weight data were assumed to be normal weight, compared to the U.S. population, by weight category and presence of ACIP-recognized chronic medical conditions***

|  | **ACIP-recognized Chronic medical conditions*** | | | | |  | **No ACIP-recognized Chronic medical conditions** | | | | |
| --- | --- | --- | --- | --- | --- | --- | --- | --- | --- | --- | --- |
|  | US Population (thousands) | Patients** | OR | 95%CI | p-value |  | US Population (thousands) | Patients** | OR | 95%CI | p-value |
|  |  |  |  |  |  |  |  |  |  |  |  |
| **2-19 Years Old** |  |  |  |  |  |  |  |  |  |  |  |
| Underweight | 214 | 14 | 8.7 | 2.2 to 35.1 | <0.001 |  | 2,146 | 11 | 3.9 | 0.8 to 18.6 | 0.1 |
| Normal weight | 6,667 | 50 | Ref | - |  |  | 39,520 | 52 | 1.0 |  |  |
| Overweight | 1,972 | 15 | 1.0 | 0.3 to 3.4 | 1.0 |  | 9,050 | 8 | 0.7 | 0.1 to 5.2 | 0.7 |
| Obese | 2,419 | 22 | 1.2 | 0.5 to 3.1 | 0.7 |  | 9,241 | 19 | 1.6 | 0.2 to 9.9 | 0.6 |
|  |  |  |  |  |  |  |  |  |  |  |  |
| **≥20 Years Old** |  |  |  |  |  |  |  |  |  |  |  |
| Underweight | 1,248 | 3 | 1.0 | 0.0 to 104 | 1.0 |  | 2,253 | 2 | 0.9 | <0.01 to 789 | 1.0 |
| Normal weight | 18,318 | 44 | Ref | - |  |  | 43,480 | 44 | 1.0 |  |  |
| Overweight | 21,316 | 28 | 0.5 | 0.3 to 1.2 | 0.1 |  | 44,412 | 15 | 0.3 | 0.1 to 1.1 | 0.1 |
| Obese | 22,527 | 44 | 0.8 | 0.4 to 1.5 | 0.5 |  | 32,495 | 21 | 0.6 | 0.3 to 1.6 | 0.3 |
| Morbidly Obese | 5,450 | 35 | 2.7 | 1.3 to 5.4 | <0.001 |  | 5,185 | 10 | 1.9 | 0.4 to 9.5 | 0.4 |

Note: OR, Odds Ratio, CI, confidence interval

BMI (kg/m^2^) categories for 2-19 year olds based on age- and sex-specific CDC 2000 growth charts: underweight<5^th^ percentile, normal weight 5-84^th^ percentile, overweight 59-94^th^ percentile, obese ≥95^th^ percentile.

BMI (kg/m^2^) categories for ≥20 year olds: underweight<18.5, normal weight 18.5-24.9, overweight 25.0-29.9, obese 30.0-39.9, morbidly obese ≥40.

* For this analysis ACIP-recognized chronic medical conditions include cardiovascular disease, pulmonary disease, liver condition, cancer, and diabetes

** Patients only included if they had an estimated weight category, and all patients with no measured height or weight data assumed to be normal weight.

**Age Adjustment**

- We combined the NHANES dataset with the hospitalized dataset. To account for the complex survey design, analysis of NHANES data requires taking into account weights strata, and primary sampling units (PSUs).
- For the hospitalized dataset we gave each patient a weight of 1 (i.e., self-weighting). We assigned all hospitalized patients to the same stratum. We randomly assigned hospitalized patients to one of two PSUs.
- We defined 4 age groups for adults so that there was an equal number of hospitalized patients in each age group (20.0-35.4, 35.5-45.9, 46.0-54.4, ≥55.0 years).
- We created a logistic regression model so that the dependent variable was whether the observation came from the hospitalized or NHANES datasets (1= hospitalized dataset, 2= NHANES). Independent variables were BMI category and age category.
- We used PROC SURVEYLOGISTIC with weights, strata, and PSUs.
- We created 4 separate logistic regression model for each level of BMI category with the Normal BMI category as the reference category (i.e., underweight vs. normal, overweight vs. normal, obese vs. normal, morbidly obese vs. normal) and ran the stratified by presence of an ACIP condition (i.e., 8 regression models in total).
- Finally, we ran 4 more logistic regression models with ACIP condition included as an independent variable.
- The first two sub-columns of Table S3 are directly comparable to Table 2 in the manuscript. It shows odds ratios (OR) for being in a specific BMI category compared to normal BMI for individuals that were hospitalized vs. from the community.
- Adjusting for age did not make much difference to the ORs for individuals with an ACIP condition or without an ACIP condition.
- The final column of Table S3 shows the OR for BMI adjusted for both age and ACIP condition. The effect of BMI remains after these adjustments.

**Table S3. Age Adjusted Odds Ratio of BMI category for patients hospitalized with 2009 pandemic influenza A(H1N1) compared to the U.S. population, by category of body mass index (BMI) and presence of ACIP-recognized chronic medical conditions***

| BMI Category | **Adjusted for Age** † | | | | | | |  | **Adjusted for Age † and ACIP-recognized Chronic medical conditions*** | | | |
| --- | --- | --- | --- | --- | --- | --- | --- | --- | --- | --- | --- | --- |
|  | ACIP-recognized Chronic medical conditions* | | |  | No ACIP-recognized Chronic Medical Conditions | | |  |  | | |  |
|  | OR | 95%CI | p-value |  | OR | 95%CI | p-value |  | OR | 95%CI | p-value |  |
| **≥20 Years Old** |  |  |  |  |  |  |  |  |  |  |  |  |
| Underweight | 1.85 | 0.77 to 4.41 | 0.17 |  | 2.32 | 0.49 to 11.01 | 0.29 |  | 2.0 | 0.63 to 6.42 | 0.24 |  |
| Normal weight | Ref | - |  |  | Ref | - | - |  | Ref | - | - |  |
| Overweight | 1.00 | 0.75 to 1.34 | 0.99 |  | 0.81 | 0.33 to 2.0 | 0.65 |  | 0.92 | 0.75 to 1.14 | 0.46 |  |
| Obese | 1.55 | 1.25 to 1.92 | <0.001 |  | 1.53 | 0.8 to 2.97 | 0.22 |  | 1.54 | 1.28 to 1.86 | <0.001 |  |
| Morbidly Obese | 4.94 | 3.30 to 7.40 | <0.001 |  | 4.58 | 3.80 to 5.53 | <0.001 |  | 4.84 | 3.53 to 6.65 | <0.001 |  |

Note: OR, Odds Ratio, CI, confidence interval.

BMI (kg/m^2^) categories for 2-19 year olds based on age- and sex-specific CDC 2000 growth charts: underweight<5^th^ percentile, normal weight 5-84^th^ percentile, overweight 59-94^th^ percentile, obese ≥95^th^ percentile.

BMI (kg/m^2^) categories for ≥20 year olds: underweight<18.5, normal weight 18.5-24.9, overweight 25.0-29.9, obese 30.0-39.9, morbidly obese ≥40.

* For this analysis ACIP-recognized chronic medical conditions include cardiovascular disease, pulmonary disease, liver condition, cancer, and diabetes

† Age group in years (20.0-35.4, 35.5-45.9, 46.0-54.4, ≥55.0)
